# Effects of habitual exercise and dietary restriction on intrahepatic and periepididymal fat accumulation in Zucker fatty rats

**DOI:** 10.1186/s13104-015-1063-6

**Published:** 2015-04-02

**Authors:** Yuka Kurosaka, Yoko Shiroya, Hideki Yamauchi, Takehiko Kaneko, Yoichiro Okubo, Kazutoshi Shibuya, Kumiko Minato

**Affiliations:** Faculty of Human Ecology, Wayo Women’s University, 2-3-1, Konodai, Ichikawa, Chiba 272-8533 Japan; Division of Physical Fitness, Department of Molecular Physiology, Jikei University School of Medicine, 8-3-1, Kokuryo, Chofu, Tokyo 182-8570 Japan; Department of Surgical Pathology, Toho University School of Medicine, 6-11-1, Omori-Nishi, Ota-Ku, Tokyo 143-8541 Japan

**Keywords:** Fatty liver, Adipocyte, Exercise, Dietary restriction, Obesity

## Abstract

**Background:**

Habitual exercise and dietary restriction are commonly recommended to prevent or ameliorate obesity and lifestyle-related diseases, including fatty liver. This study investigated the effects of habitual exercise and dietary restriction on hepatic triglyceride (TG) levels, serum leptin levels, and histological adipocyte size in periepididymal adipose tissue from Zucker fatty (ZF) rats.

**Methods:**

Six-week-old male ZF rats were randomly assigned to one of three groups: sedentary (Sed), sedentary and dietary restriction (Sed + DR), and training and dietary restriction (Tr + DR). Male Zucker lean (L) rats were used as control animals. All rats had access to water and the allowed quantity of food *ad libitum*. The rats in the Sed + DR and Tr + DR groups were fed a 30% restricted diet, while those in the Tr + DR group exercised voluntarily on a wheel ergometer. After 12 weeks, the rats were sacrificed for a histological examination of their liver and periepididymal adipose tissue. Hepatic and serum TG, serum total cholesterol, glutamic-oxaloacetic transaminase, glutamic-pyruvic transaminase, free fatty acid, and leptin levels were also measured.

**Results:**

The hepatic TG levels were significantly higher in the Sed + DR group than in the L (*P* < 0.001) and Sed (*P* < 0.05) groups. By contrast, the hepatic TG levels in the Tr + DR group were significantly lower than those in the Sed (*P* < 0.05) and Sed + DR (*P* < 0.001) groups, but not significantly different from the L group values. The periepididymal adipocytes were significantly larger in the Sed, Sed + DR, and Tr + DR groups than in the L group (*P* < 0.001) and were significantly smaller in the Tr + DR group compared to the Sed and Sed + DR groups (*P* < 0.001).

**Conclusions:**

Our results suggest a relationship between lipid metabolism and the size of adipose cells in ZF rats. Exercising plays an important role in decreasing hepatic TG levels, serum leptin levels, and the size of adipose cells.

## Background

Although obesity increases the risk of fatty liver disease, hepatic fat accumulation can also develop in non-obese individuals [1,2]. Furthermore, the conditions associated with severe malnutrition (e.g., kwashiorkor) can cause fatty liver disease [3]. These observations suggest that excessive food intake and poor nutrition contribute to fat accumulation in the liver. In general, habitual exercise and dietary modification have an inhibitory effect on obesity [4,5]. Previous large-scale cohort studies found that physical fitness as assessed by muscle strength and endurance, body mass index (BMI), and fat percentage influenced the health profile of individuals [6-8]. Moreover, studies suggest that exercise has an effect on morbidity and mortality from lifestyle-related diseases. For instance, individuals with elevated BMI values who engage in habitual exercise have been found to have a lower mortality rate than those with a normal BMI who do not exercise regularly [6-8]. Furthermore, a lack of habitual exercise is equivalent to smoking as a risk for noncommunicable disease [9].

Both habitual exercise and dietary restriction contribute to weight loss. However, few reports have examined these effects simultaneously. We recently characterized the effects of habitual exercise and/or diet restriction on hepatic fat accumulation in Zucker fatty (ZF) rats [10]. Histologically, the intrahepatic accumulation of triglycerides (TGs) was suppressed by habitual exercise, but not by dietary restriction alone [10]. ZF rats have a mutation in the leptin receptor gene that causes increased appetite and weight gain; thus, they are used as an animal model of obesity [11].

We used ZF rats to examine the effects of habitual exercise and dietary restriction on hepatic TG levels, serum leptin levels, and histological adipocyte size in periepididymal adipose tissue.

## Methods

### Animals

Our study was approved by the Biological and Epidemiologic Research Committee for Animal Use of Wayo Women’s University (Ichikawa, Japan). All procedures were performed in accordance with the Declaration of Helsinki. Male Zucker lean (L) rats and male ZF rats were purchased at 5 weeks of age (87.0–100.9 and 118.4–138.0 g each, respectively) from Charles River Laboratories Japan, Inc. (Yokohama, Japan). All animals were housed in individual cages in a temperature-controlled (21.8 ± 0.6°C) animal room with a 12-h light (08:00–20:00)/dark (20:00–08:00) cycle. Following a 1-week acclimation period, the ZF rats, aged 6 weeks, were allocated to one of three groups using computer-generated randomization: sedentary (Sed), sedentary + dietary restriction (Sed + DR), or training + dietary restriction (Tr + DR). All animals received standard rodent chow (NMF; Oriental Yeast Co., Ltd., Tokyo, Japan) and were allowed *ad libitum* access to water and the allowed quantity of food. Body weight and food consumption were monitored daily throughout the study period. The animals in the Sed + DR group received 70% of the mean volume of food consumed by the animals in the Sed group. The animals in the Tr + DR group were subjected to a dietary restriction but were allowed unrestricted access to a running wheel. Running distance was measured by an ergometer; the mean distance was 2,533 m/24 h. Furthermore, the quantity of food was regulated to allow equivalent weight gain in the Tr + DR and Sed + DR groups. L rats, a non-hyperphagic strain used as a control, were maintained under sedentary conditions.

At 12 weeks of age, all animals were fasted overnight to exclude the influence of diet up until the day before and the Tr + DR running wheels were locked. The rats were anesthetized with isoflurane, and blood samples were collected into serum gel separator tubes (VENOJECT II plastic vacuum tube; Terumo, Tokyo, Japan) and centrifuged to separate the serum for blood chemistry tests. The liver and periepididymal adipose tissue were rapidly resected from the anesthetized rats. Samples of adipose tissue and hepatic tissue were placed in 10% formalin, and the remaining tissues were frozen in liquid nitrogen and stored at −80°C until analysis. The rats were then sacrificed by fatal exsanguination.

### Serum analysis

Serum levels of free fatty acids (FFA), leptin, TG, total cholesterol (T-CHO), high-density lipoprotein cholesterol (HDL-C), glutamic-oxaloacetic transaminase (GOT), and glutamic-pyruvic transaminase (GPT) were measured using commercially available kits according to the manufacturer’s instructions (FFA: NEFA E-test, Wako Pure Chemical Industries Ltd., Osaka, Japan; leptin: YK050 Rat Leptin ELISA Kit, Yanaihara Institute, Shizuoka, Japan; TG: TG E-test, Wako Pure Chemical Industries Ltd.; T-CHO, HDL-C, GOT, and GPT: Dri-Chem 4000, Fujifilm, Tokyo, Japan).

### Liver tissue preparation for TG analysis

To extract lipids, the resected liver was homogenized with a 2:1 chloroform-methanol mixture (vol/vol) [12]. Hepatic TG levels were determined using a kit (TG E-Test, Wako Pure Chemical Industries Ltd.).

### Histological examination

Resected hepatic and periepididymal adipose tissues were fixed in 10% formalin. Hematoxylin and eosin (H&E) staining was outsourced to SRL (Tokyo, Japan). Photomicrographs of the sections were made for digital storage. Adipocyte size was determined by measuring the cross-point intervals as described previously [13,14]. Briefly, more than 2,000 parallel lines were superimposed onto the tissue sections of three animals using ImageJ software (version 1.46, http://rsb.info.nih.gov/ij/; National Institutes of Health, Bethesda, MD, USA). Then, the distances between the intersections of the lines and the cell membranes were measured. All ambiguous structures (e.g., vascular structures and the parallel lines touching the edges of the image fields) were excluded from analysis.

### Statistical analyses

A one-way analysis of variance was performed for each outcome measure. Significant main effects were followed up using Fisher least-significant difference post hoc comparisons. All values are expressed as means ± standard error of the mean (SEM); *P*-values < 0.05 were deemed to indicate statistical significance.

## Results

### Animal characteristics

At 12 weeks of age, the rats in the Sed (*P* < 0.001), Sed + DR (*P* < 0.05), and Tr + DR (*P* < 0.05) groups weighed significantly more than the animals in the L group. The rats in the Sed group also weighed significantly more than those in the Sed + DR and Tr + DR groups (both, *P* < 0.001); however, body weight was not significantly different between the Sed + DR and Tr + DR groups. Mean daily food intake was significantly greater in the Sed, Sed + DR, and Tr + DR groups than in the L group (*P* < 0.001) and significantly less in the Sed + DR and Tr + DR groups than in the Sed group (*P* < 0.001); however, mean daily food intake did not differ between the Sed + DR and Tr + DR groups. The wet weight of the liver was significantly greater in the Sed (*P* < 0.001) and Sed + DR (*P* < 0.05) rats compared to the L group and significantly less in the Tr + DR than in the Sed group (*P* < 0.01). Moreover, the weight of the soleus muscle was significantly lower in the Sed, Sed + DR, and Tr + DR groups than in the L group (*P* < 0.001), but significantly higher in the Tr + DR than in the Sed and Sed + DR groups (*P* < 0.001; Table 1).

**Table 1:** **Animal characteristics**

**Characteristics**	**Study groups**			
	**L**	**Sed**	**Sed + DR**	**Tr + DR**
Body weight, g	299.2 ± 6.0	447.3 ± 11.9***	341.0 ± 7.5*^§§§^	339.6 ± 11.5*^§§§^
Food intake, g/day	20.8 ± 0.5	34.3 ± 0.7***	24.4 ± 0.1***^§§§^	24.5 ± 0.6***^§§§^
Running distance, m/day	NS	NS	NS	2553 ± 425
Liver weight, mg/g BW	31.6 ± 0.7	51.3 ± 3.4***	42.6 ± 3.9*	36.9 ± 1.0^§§^
Soleus muscle weight, mg/100 g BW	43.2 ± 0.6	28.1 ± 1.1***	24.8 ± 0.8***	36.3 ± 1.2***^§§§†††^

All values are expressed as the mean ± SEM (*n* = 6 per group). *P < 0.05 and ***P < 0.001 vs. the L group. ^§§^ *P* < 0.01 and ^§§§^ *P* < 0.001 vs. the Sed group. ^†††^ *P* < 0.001 vs. the Sed + DR group. L, lean control group; Sed, sedentary group; Sed + DR, sedentary + dietary restriction group; Tr + DR, training + dietary restriction group; NS, not significant.

### Hepatic fat levels

**Figure 1:**
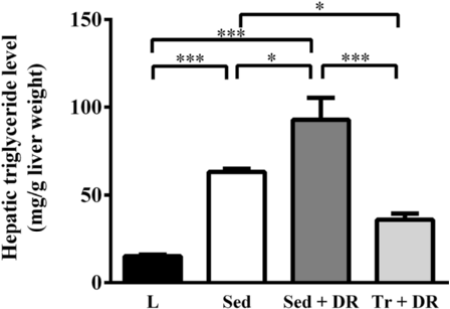
**Effects of habitual exercise and dietary restriction on hepatic triglycerides.** Hepatic triglyceride levels in the lean (L), sedentary (Sed), sedentary and dietary restriction (Sed + DR), and training and dietary restriction (Tr + DR) groups. All values are expressed as the mean ± standard error of the mean (SEM; *n* = 6 per group). *P < 0.05; ***P < 0.001.

**Figure 2:**
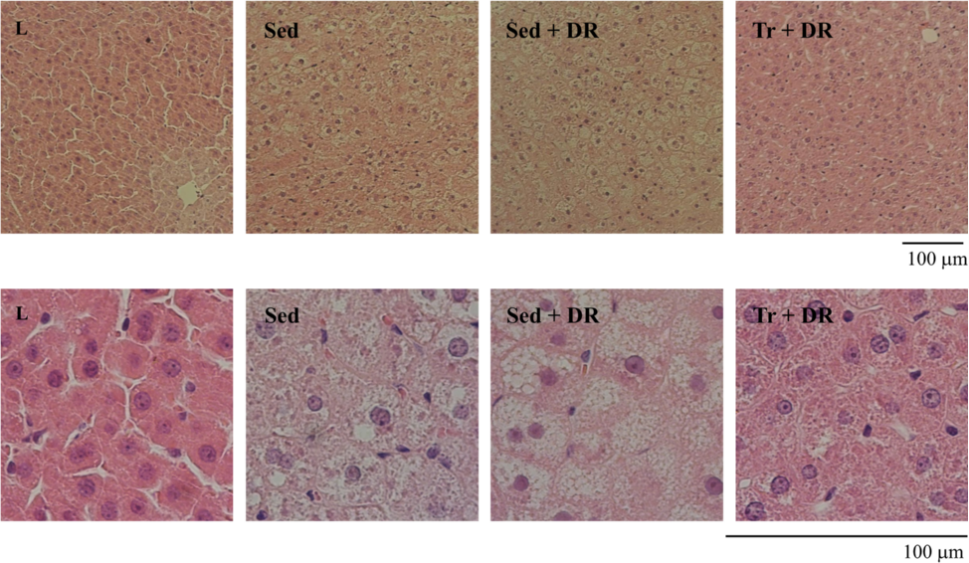
**Representative photomicrographs of hematoxylin and eosin (H&E)-stained hepatic tissue samples.** Note the fat infiltration in the sedentary (Sed), sedentary and dietary restriction (Sed + DR) groups.

Hepatic TG levels were significantly higher in the Sed + DR group than in the L (*P* < 0.001) and Sed (*P* < 0.05) groups. In contrast, the hepatic TG levels in the Tr + DR group were significantly lower than those in the Sed (*P* < 0.05) and Sed + DR (*P* < 0.001) groups, but not significantly different from the L group values (Figure 1). Representative photomicrographs of H&E-stained hepatic tissue samples are shown in Figure 2.

### Serum chemistry

**Figure 3:**
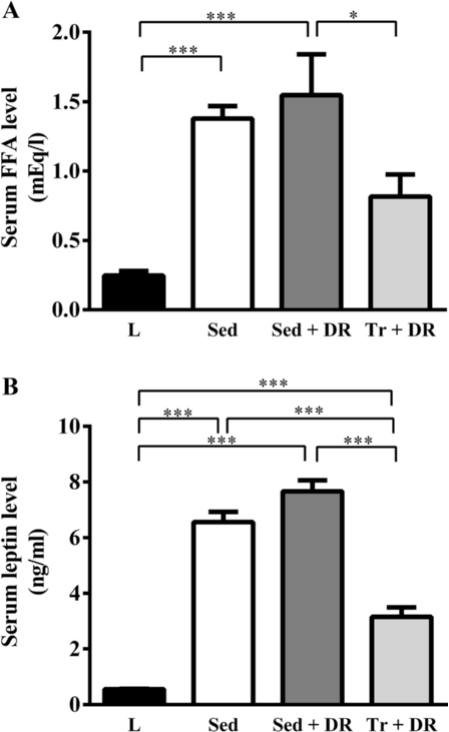
**Effects of habitual exercise and dietary restriction on serum free fatty acid (FAA) and leptin levels.** Serum levels of free FFAs **(A)** and leptin **(B)** in the lean (L), sedentary (Sed), sedentary and dietary restriction (Sed + DR), and training and dietary restriction (Tr + DR) groups. All values are expressed as the mean ± SEM (*n* = 6 per group). *P < 0.05; ***P < 0.001.

The results of serum chemical analyses are shown in Table 2 and Figure 3. The serum FFA levels were significantly higher in the Sed and Sed + DR groups than in the L group (*P* < 0.001), and the Sed + DR group levels were higher than those in the Tr + DR group (*P* < 0.05; Figure 3). The serum leptin levels were higher in the Sed, Sed + DR, and Tr + DR groups than in the L group (*P* < 0.001), and the serum leptin levels in the Sed and Sed + DR groups were higher than those in the Tr + DR group (*P* < 0.001; Figure 3). The serum TG levels were significantly higher in the Sed (*P* < 0.001) and Sed + DR (*P* < 0.01) groups than in the L group (Table 2). Serum T-CHO was significantly higher in the Sed and Sed + DR groups than in the L group (*P* < 0.001), and higher in the Sed (*P* < 0.05) and Sed + DR (*P* < 0.001) groups than in the Tr + DR group (Table 2). Serum HDL-C was significantly higher in the Sed + DR group than in the L (*P* < 0.001), Sed + DR (*P* < 0.05), and Tr + DR (*P* < 0.001) groups (Table 2). Moreover, the serum HDL-C levels were significantly higher in the Sed than in the L (*P* < 0.001) and Tr + DR (*P* < 0.05) groups (Table 2). Serum GOT was significantly higher in the Sed + DR than in the L group (*P* < 0.05), and serum GPT levels were significantly higher in the Sed + DR than in the L and Tr + DR groups (both, *P* < 0.05; Table 2).

**Table 2:** **Serum chemistry**

**Characteristics**	**Study groups**			
	**L**	**Sed**	**Sed + DR**	**Tr + DR**
Triglycerides, mg/100 mL	114.2 ± 36.3	736.6 ± 135.4***	585.7 ± 81.5**	420.6 ± 57.0
Total cholesterol, mg/100 mL	54.3 ± 1.8	113.2 ± 9.5***	128.3 ± 13.7***	72.8 ± 1.8^§†††^
HDL cholesterol, mg/100 mL	43.2 ± 1.1	73.0 ± 4.9***	91.3 ± 6.7***^§§§^	56.2 ± 0.7^§†††^
GOT, U/L	61.7 ± 2.5	162.7 ± 31.5	229.0 ± 76.3*	83.5 ± 10.9
GPT, U/L	32.8 ± 4.3	84.8 ± 21.4	168.2 ± 55.5*	45.7 ± 7.8^†^

All values are expressed as the mean ± SEM (*n* = 6 per group). *P < 0.05; **P < 0.01; and ***P < 0.001 vs. the L group. §, *P* < 0.05 and ^§§§^ *P* < 0.001 vs. the Sed group. ^†^ *P* < 0.05 and ^†††^ *P* < 0.001 vs. the Sed + DR group. HDL, high-density lipoprotein; GOT, glutamic-oxaloacetic transaminase; GPT, glutamic-pyruvic transaminase; L, lean; Sed, sedentary; Sed + DR, sedentary + dietary restriction; Tr + DR, training + dietary restriction; NS, not significant.

### Periepididymal adipose tissue

**Figure 4:**
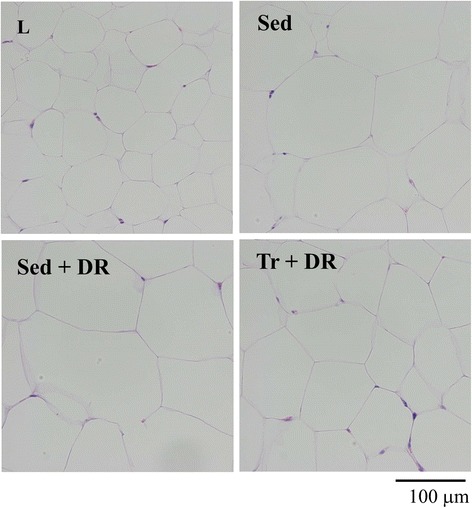
**Representative photomicrographs of hematoxylin and eosin (H&E)-stained periepididymal adipose tissue samples.** Adipocyte hypertrophy was observed in the sedentary (Sed) and sedentary and dietary restriction (Sed + DR) groups, but not in the lean (L) and training and dietary restriction (Tr + DR) groups.

**Figure 5:**
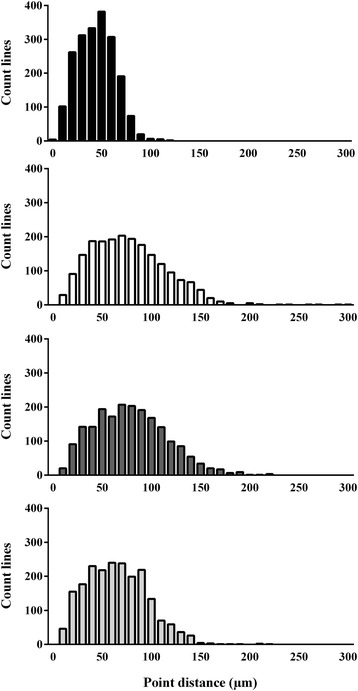
**Distribution of periepididymal adipocyte size.** Distribution of periepididymal adipocyte size in the lean (L), sedentary (Sed), sedentary and dietary restriction (Sed + DR), and training and dietary restriction (Tr + DR) groups. Adipocyte size was measured 2,000 times in each group (*n* = 3).

**Figure 6:**
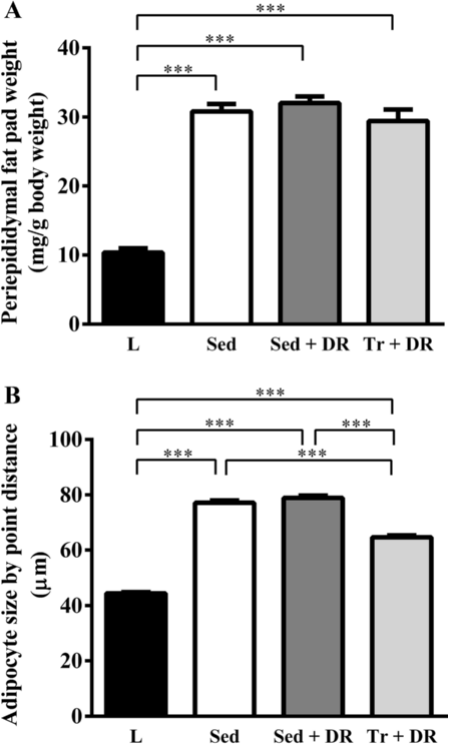
**Effects of habitual exercise and dietary restriction on periepididymal fat wet weights and adipocyte size.** Periepididymal fat wet weights **(A)** and adipocyte size by point distance **(B)** in the L, Sed, Sed + DR, and Tr + DR groups. Adipocyte size was measured 2,000 times in each group (*n* = 3). ***P < 0.001.

Representative photomicrographs of H&E-stained periepididymal adipose tissue, adipocyte size distribution, and periepididymal fat pad weight and adipocyte sizes are shown in Figures 4, 5 and 6, respectively. The mean periepididymal fat pad weight was significantly higher in the Sed, Sed + DR, and Tr + DR groups than in the L group (*P* < 0.001); however, the weight was not significantly different among the Sed, Sed + DR, and Tr + DR groups (Figure 6). The periepididymal adipocyte size was significantly larger in the Sed, Sed + DR, and Tr + DR groups than in the L group and larger in the Sed and Sed + DR groups than in the Tr + DR group (all, *P* < 0.001; Figure 6).

## Discussion

To our knowledge, this is the first study to examine the effects of habitual exercise and dietary restriction on adipocyte size in periepididymal adipose tissue. The original goal of this study was to make a direct comparison of the effect on fatty liver disease between the restricted diet group and the restricted diet plus exercise group.

We found no significant difference in food consumption (i.e., consumed calories) between the Sed + DR and Tr + DR groups. Moreover, body weight and periepididymal fat weight were similar between these groups after 12 weeks. However, the hepatic TG levels and periepididymal adipocyte size were significantly lower in the Tr + DR than in the Sed + DR group. These data indicate positive effects of exercise on hepatic TG levels and adipocyte size in the epididymis.

In this study, despite the fact that body weight gain was suppressed by dietary restriction as expected, hepatic fat accumulation was greatly facilitated. On this point, we considered the possibility that the metabolic state of the animals in the Sed + DR group was similar to that during starvation. ZF rats are affected by overeating, and the reported level of overeating is great [15]. Therefore, we considered that the metabolic state of the ZF rats was probably close to the starvation state.

From the hepatic fat accumulation data, we speculated that the suppression of serum FFA with exercise induced the suppression of hepatic fat accumulation mediated by a decrease in fatty acid uptake into hepatocytes. In addition, regarding the increase in serum FFA caused by dietary restriction only, we speculated that lipolysis, which occurs in fat cells during severe starvation, occurred in the Sed + DR group. We also postulated that the result of the increased hepatic fat accumulation in the Sed + DR group was induced by increased fatty acid uptake into hepatocytes, which was caused by the increased serum level of FFA. Indeed, previous studies suggested that high levels of FFA are secreted from large size of fat cell [16,17].

Hoffstedt et al. [18] compared obese patients with a few large adipocytes (adipose hypertrophy) and those with many small adipocytes (adipose hyperplasia) and found that patients with many small adipocytes had healthier blood glucose, insulin, and lipid level profiles than did patients with large adipocytes. We found that the serum T-CHO, HDL-C, FFA, GOT, and GPT levels were lower in the Tr + DR group than in the Sed + DR group. Our results also suggest a relationship between lipid metabolism and adipocyte size. In general, the conditions that cause excessive fat accumulation (i.e., fatty liver) are related to the development of insulin resistance and various internal organ dysfunctions [19]. The suppression of adipocyte hypertrophy is said to be an important factor in maintaining healthy lipid metabolism.

In a study of Wistar rats subjected to exercise, Miyazaki et al. [20] found that exercise reduced leptin mRNA expression and the size of adipocytes in adipose tissue, and they concluded that the exercise-induced change in leptin levels was dependent on a reduction in adipocyte size. Similar changes were observed in morbidly obese ZF rats. Noto et al. [21] reported a decrease in both serum leptin levels and the size of adipose cells after the intake of conjugated linoleic acid. Our findings indicate a significant decrease in serum leptin levels and adipocyte size in the Tr + DR but not in the Sed + DR group (Figures 3 and 6), consistent with these previous findings [20,21], and suggest an association between leptin levels and adipocyte size in the exercise group. If changes in adipocyte size can induce hepatic fat accumulation, then adipocytes maintained in good condition could prevent hepatic fat accumulation with habitual exercise.

## Conclusions

Our results suggest that a relationship exists between lipid metabolism and the size of adipose cells in ZF rats. Exercise plays an important role in decreasing hepatic TG levels, serum leptin levels, and the size of adipose cells.

## Abbreviations

BMI: Body mass index
TG(s): Triglyceride(s)
ZF: Zucker fatty
L: Zucker lean
Sed: Sedentary
Sed + DR: Sedentary + dietary restriction
Tr + DR: Training + dietary restriction
FFA: Free fatty acid
T-CHO: Total cholesterol
HDL-C: High-density lipoprotein cholesterol
GOT: Glutamic-oxaloacetic transaminase
GPT: Glutamic-pyruvic transaminase
H&E: Hematoxylin and eosin
SEM: Standard error of the mean
